# From Revisions to Insights: Converting Radiology Report Revisions into Actionable Educational Feedback Using Generative AI Models

**DOI:** 10.1007/s10278-024-01233-4

**Published:** 2024-08-19

**Authors:** Shawn Lyo, Suyash Mohan, Alvand Hassankhani, Abass Noor, Farouk Dako, Tessa Cook

**Affiliations:** Department of Radiology, Hospital of the University of Pennsylvania, Philadelphia, PA USA

**Keywords:** Radiology training, Generative artificial intelligence, Large language models, Report revisions, Education, Precision radiology education

## Abstract

Expert feedback on trainees’ preliminary reports is crucial for radiologic training, but real-time feedback can be challenging due to non-contemporaneous, remote reading and increasing imaging volumes. Trainee report revisions contain valuable educational feedback, but synthesizing data from raw revisions is challenging. Generative AI models can potentially analyze these revisions and provide structured, actionable feedback. This study used the OpenAI GPT-4 Turbo API to analyze paired synthesized and open-source analogs of preliminary and finalized reports, identify discrepancies, categorize their severity and type, and suggest review topics. Expert radiologists reviewed the output by grading discrepancies, evaluating the severity and category accuracy, and suggested review topic relevance. The reproducibility of discrepancy detection and maximal discrepancy severity was also examined. The model exhibited high sensitivity, detecting significantly more discrepancies than radiologists (*W* = 19.0, *p* < 0.001) with a strong positive correlation (*r* = 0.778, *p* < 0.001). Interrater reliability for severity and type were fair (Fleiss’ kappa = 0.346 and 0.340, respectively; weighted kappa = 0.622 for severity). The LLM achieved a weighted F1 score of 0.66 for severity and 0.64 for type. Generated teaching points were considered relevant in ~ 85% of cases, and relevance correlated with the maximal discrepancy severity (Spearman *ρ* = 0.76, *p* < 0.001). The reproducibility was moderate to good (ICC (2,1) = 0.690) for the number of discrepancies and substantial for maximal discrepancy severity (Fleiss’ kappa = 0.718; weighted kappa = 0.94). Generative AI models can effectively identify discrepancies in report revisions and generate relevant educational feedback, offering promise for enhancing radiology training.

**Supplementary Information:**

The online version contains supplementary material available at 10.1007/s10278-024-01233-4.

## Introduction

Delivering expert feedback on trainees’ preliminary reports is a foundational aspect of radiologic training. Traditionally, this feedback is given during readouts at the workstation at approximately the time of dictation [1]. However, shifts where trainees and faculty read non-contemporaneously (such as overnight shifts), remote reading and the escalating volumes of imaging studies are factors that reduce the frequency of this direct synchronous feedback [2, 3]. Furthermore, as trainees become more experienced, some may prefer increased autonomy and more time spent dictating compared to reading out [1].

Revisions to trainee reports in the dictation system include the trainee’s preliminary report and a finalized report generated by an attending after their review. These revisions may be the only source of feedback for reports where readouts are not feasible and also contain data with substantial educational potential [2]. Data within revisions can reveal insights into areas where trainees can improve their search patterns, knowledge of anatomy, pathology, imaging artifacts, integration of clinical information, reporting techniques, and more. However, accessing and synthesizing the feedback from revisions can be arduous for trainees.

Reviewing each revision individually can be a time-consuming, potentially low-yield, and error-prone process [2, 4]. Prior efforts have been made toward improving the accessibility and functionality of report revisions. O’Connell and Chang [5] created a report viewer integrated with PACS that displayed preliminary and finalized reports side by side. Sharpe et al. [2] collated report revisions over several reports, which improved the convenience of accessing multiple revisions. Chen et al. [6] created an automated case log system that logs preliminary and finalized reports with an interface that flags reports containing clinically significant addenda as defined by report macros. Kalaria et al. [4] implemented a system where weekly reports containing highlighted changes between preliminary and finalized reports were emailed to trainees. Durojaye et al. [7] created a system that integrated case volumes and feedback with other metrics to provide overall trainee assessments. These efforts represented significant incremental advances in increasing the ease of accessing revision data and integration into trainee assessment. However, there remains a gap in that the raw revision data lacks a structured targeted educational format convenient for synthesis and learning.

With the advent of large language models (LLMs), there is significant potential to fill this gap and transform raw revision data into targeted educational feedback. LLMs are a class of neural networks that are trained on large datasets and are used to process human language [8, 9]. LLMs are strong at natural language processing tasks, including extracting or classifying text [10]. They can synthesize data [8, 11] and have demonstrated the capacity to parse radiology reports [12]with numerous other emerging applications within radiology [13–16]. Notably, Gertz et al. [17] demonstrated that LLMs could identify common errors in radiology reports using single zero-shot prompting instances with a detection rate that was comparable to that of radiologists.

We hypothesized that an LLM could utilize report revisions to accurately identify discrepancies in preliminary reports, grade the severity, and classify the type of discrepancy across distinct predefined categories. Additionally, the data from these discrepancies can be synthesized to recommend topics for trainees to review which are specifically tailored to their reports, representing a crucial step toward tailoring education to a trainee’s overall needs in this coming age of “AI-augmented precision radiology education” [18, 19].

## Materials and Methods

### Model Selection and Deployment

We selected the OpenAI GPT-4 Turbo API (GPT4-1104-preview) to compare paired synthetic and open-source analogs of preliminary and finalized radiology reports. The model was chosen due to its context window of 128,000 tokens, which was suited to the length and content of radiology reports and our intended prompts. A preliminary analysis of report token length was performed using the Python tiktoken library, and a maximal token length of 1865 tokens was demonstrated for a radiology report. In conjunction with our prompts, which combined preliminary and final reports, the maximum total prompt input token length ranged up to 3585 tokens, which neared the 4096 input token limit of GPT-3.5. Input size relative to context window length has also been described as one factor contributing to output incoherence or degradation [20, 21]. Preliminary testing of prompts with GPT-3.5 showed numerous instances where the model produced incoherent output (e.g., “Sorry, as a large language model, I am unable to analyze radiology reports”). A temperature setting of 0 was chosen to promote determinism in data output [22]. The API was called from within an application built using PHP 7.1 and MySQL, hosted on an Ubuntu Linux server running Apache 2.0.

### Datasets

Our institution prohibits the use of deidentified reports with public OpenAI, and we did not have access to a closed OpenAI instance. The “preliminary” and “finalized” radiology reports were obtained from two external datasets. The first dataset comprised 50 pairs of neuroradiology reports, which were synthesized using OpenAI GPT-4. The synthesized reports were created using a prompt instructed to randomly generate either no significant discrepancies or reports with multiple discrepancies of varying degrees of severity, which are described in Table 1. Neuroradiology reports were chosen as they aligned with our expert raters’ subspecialties. The second dataset contained 50 pairs of reports from the ReXVal dataset, which included generated report impressions, ground truth report impressions derived from the MIMIC-CXR dataset, and expert radiologist-annotated discrepancies between the generated and ground truth reports [23]. This dataset was chosen because it included expert annotations of both total and clinically significant discrepancies. The full dataset of reports utilized in this study is available in Appendix B. An Institutional Review Board (IRB) exemption was obtained.

**Table 1:** Discrepancy severity and type classification categories and their descriptions

Classification task	Category	Description supplied in the prompt
Severity	Major	Discrepancy that could cause immediate or irreparable harm to patients such as missed pneumothorax, pulmonary embolism, acute intracranial hemorrhage, large vessel occlusion, bowel perforation…
	Minor	Discrepancy that may affect clinical treatment but without significant potential for immediate harm, such as favoring a different etiology within a list of differentials…
	Addition	Discrepancy which adds more detail but may not necessarily affect clinical treatment
	Stylistic	Discrepancy which changes only the phrasing and not the content of the report
	Other	Discrepancies not in the above types
Type	Perceptual	Finding that was not seen
	Interpretive	Finding that was seen and misinterpreted
	Typographic	Typographic
	Other	Discrepancies not in the above types

These definitions were adapted from previous discrepancy severity and type classifications and slightly modified for prompt performance [24–26]

### Model Prompts and Outputs

#### Discrepancy Detection

**Fig. 1:**
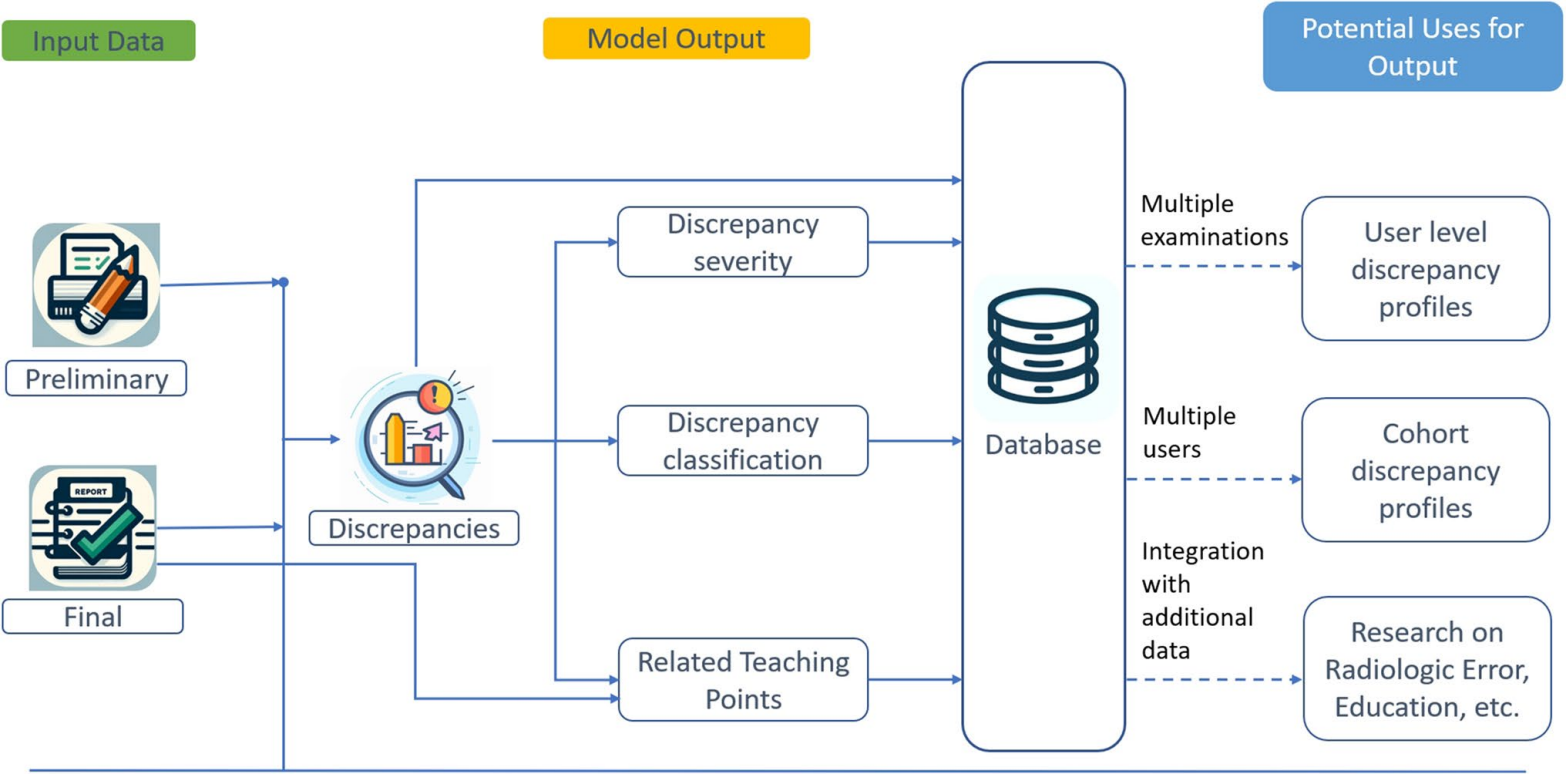
Workflow diagram for discrepancy analysis*.* Preliminary and finalized reports are compared, and discrepancies are identified. The discrepancies are then analyzed and classified by severity and category. The discrepancy analysis is then used to generate teaching points related to the report. The generated data can be used for further analyses

**Fig. 2:**
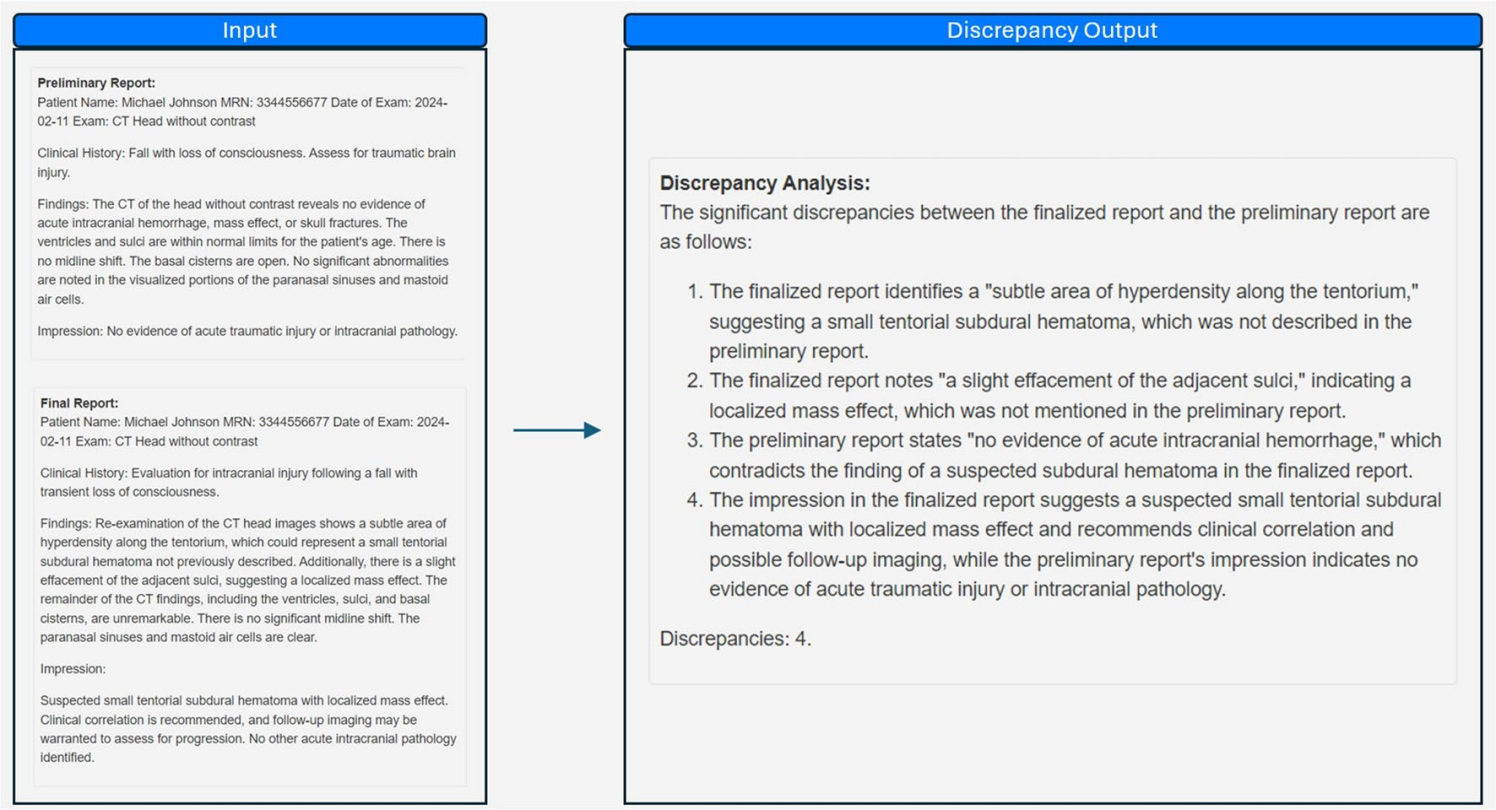
Generating discrepancy output from paired preliminary and finalized reports. The preliminary and finalized reports (left) serve as inputs for a prompt, which produces the discrepancy output (right). The discrepancy output identifies and details discrepancies detected by the AI model and provides a total count of discrepancies

Using paired preliminary and finalized reports as inputs, a zero-shot prompt utilizing role, context, conditional, and output instruction (Appendix A) generates the discrepancy detection output, which contains the total number of discrepancies and a description of each discrepancy (Figs. 1 and 2).

#### Discrepancy Classification

**Fig. 3:**
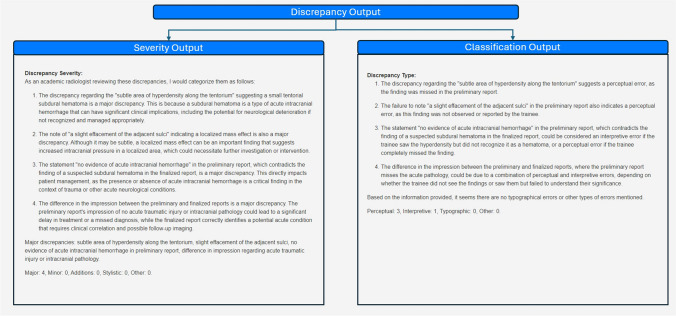
Generating severity and type classification outputs from discrepancy output. The discrepancy output serves as an input for prompts that generate assessments of severity and type for each identified discrepancy and provide the number of discrepancies of each type

The list of discrepancies serves as input for a subsequent set of one-shot and few-shot prompts utilizing role, context, conditional, output instructions, and elements of thought generation/zero-shot chain of thought (CoT) (Figs. 1 and 3 and Appendix A), which further classifies the discrepancies based on severity and type. Severity analysis classifies discrepancies as major, minor, addition, stylistic, or other, while type analysis classifies discrepancies as perceptual, interpretive, typographic, or other (Table 1 and Fig. 3). The severity categories and criteria were modeled after attending discrepancy feedback report attestation macros, currently utilized at our institution. Both severity and classification outputs contain descriptive assessments of each discrepancy and report the number of discrepancies of each type.

#### Topics for Review

**Fig. 4:**
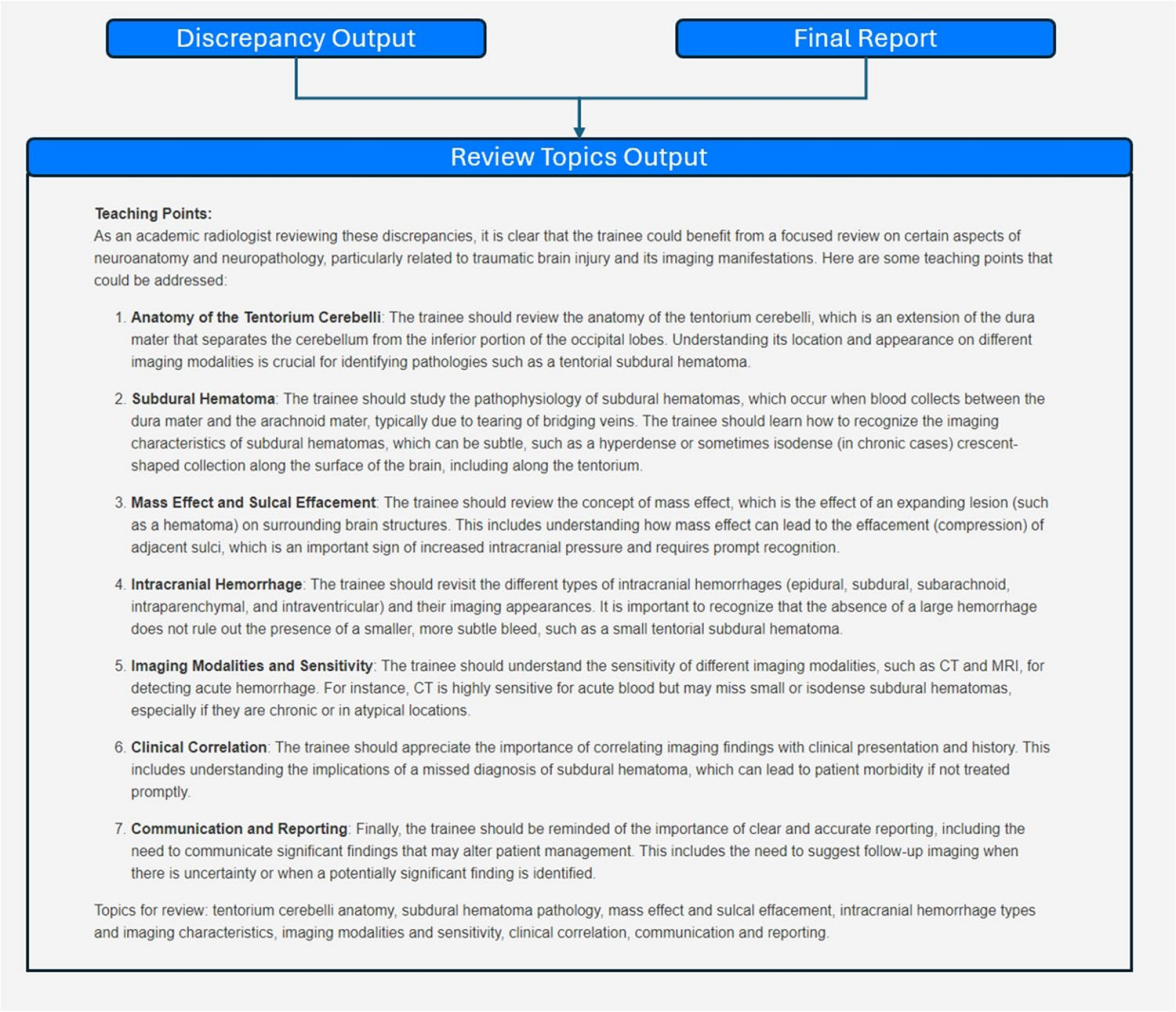
Generating topics for review. The discrepancy output and the finalized report are inputs for a prompt that produces a list of topics for trainees to review

The list of discrepancies identified by the LLM and the final report serves as inputs to a zero-shot prompt utilizing role, context, output, and conditional instruction with elements of thought generation/zero-shot CoT, which generates a list of suggested topics beneficial for trainee review (Appendix A). The output includes a list of teaching points with associated explanations and a final comma-delimited list of topics (Fig. 4).

### Output Grading

#### Synthesized Neuroradiology Reports

The model outputs from the analysis of the synthesized preliminary and final neuroradiology reports were rated by three neuroradiology subspecialty-trained radiologists in randomized order. The radiologists indicated the number of discrepancies they identified, the number of discrepancies identified by expert radiologists that the LLM did not recognize, and the number of discrepancies identified by the LLM that were incorrectly identified. For this study, discrepancies that were redundant or related to differences in wording were not considered false.

Each discrepancy identified was then classified by severity and type using the same categories provided by the LLM. For severity classification, we made a philosophical choice to compare model performance to current clinical practice. Radiologists were not provided definitions but asked to apply their implicit understanding of the severity terms based on how they had been utilizing them in practice [24]. For discrepancy-type classification, raters were instructed to classify errors that were not detected as “perceptual errors” and those that were detected but misinterpreted as “interpretive (or cognitive) errors” [26].

The teaching points generated by the LLM were rated based on their perceived relevance to the case on a 1–5 Likert scale where 1 was “not relevant,” and 5 was “extremely relevant.”

#### RexVal Dataset

The RexVal dataset contained expert ratings concerning the total number of discrepancies and the number of clinically significant discrepancies between reports. The clinically significant discrepancies were considered analogous to our classification’s severity ratings of major and minor discrepancies. The number of total and clinically significant discrepancies were compared to the outputs of our LLM.

#### Reproducibility Analysis

Following the initial analysis, we assessed the outputs’ reproducibility. Given the chained nature of outputs in this study, the number of discrepancies identified and the maximal severity of discrepancies identified within a report were the variables that most lent themselves to reliability analysis. We repeated the inferencing from the discrepancy detection and severity classification prompts five times for each report and analyzed the reliability of the number of discrepancies and maximal severity identified.

### Statistical Analysis

Statistical analysis was performed utilizing Python 3.10.12 and libraries for data analysis such as Pingouin 0.5.4, SciPy 1.13.1, and scikit-learn 1.3.2. The analysis included multiple univariate and multivariate tests as appropriate. Wilcoxon rank-sum and correlation were used to compare the numbers of discrepancies identified by raters and by the LLM. Fleiss’ kappa was used to assess interrater reliability and agreement, first between radiologists alone and then with a combined group including radiologists and the LLM. Given that the severity ratings contained an ordinal aspect, quadratic weighted kappa was also performed. The performance of the LLM on classification tasks was assessed utilizing precision, recall, and F1 score using a probabilistic model of agreement with each individual expert rating [27]. Precision, recall, and F1 scores were also performed on the subset of discrepancies with uniform agreement in expert ratings. Correlations were utilized to assess the relationships of numerous variables as appropriate. Reproducibility analysis was performed using the coefficient of variation and intraclass correlation coefficient (ICC (2,1)) for the number of discrepancies and Fleiss’ kappa and quadratic weighted kappa for maximal discrepancy severity. A *p*-value of 0.05 was set as the threshold for statistical significance.

## Results

### Discrepancy Detection and Accuracy

**Fig. 5:**
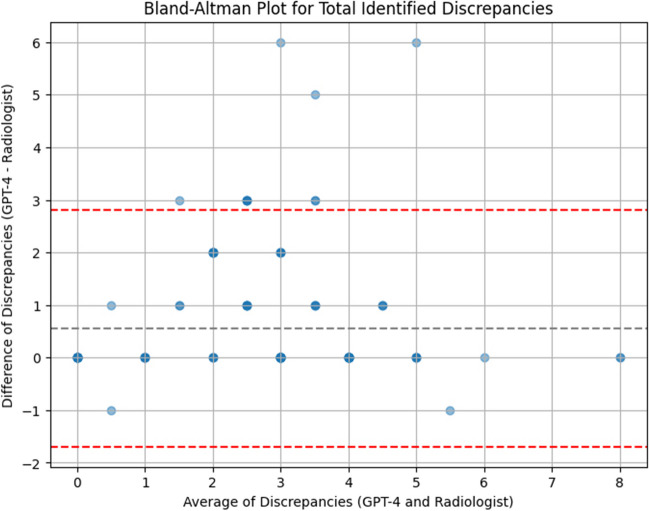
Bland–Altman plot demonstrating agreement between the number of discrepancies identified by radiologists and the number of discrepancies identified by LLM. Radiologists and LLM had a moderately significant positive correlation (*r* = 0.778)

**Fig. 6:**
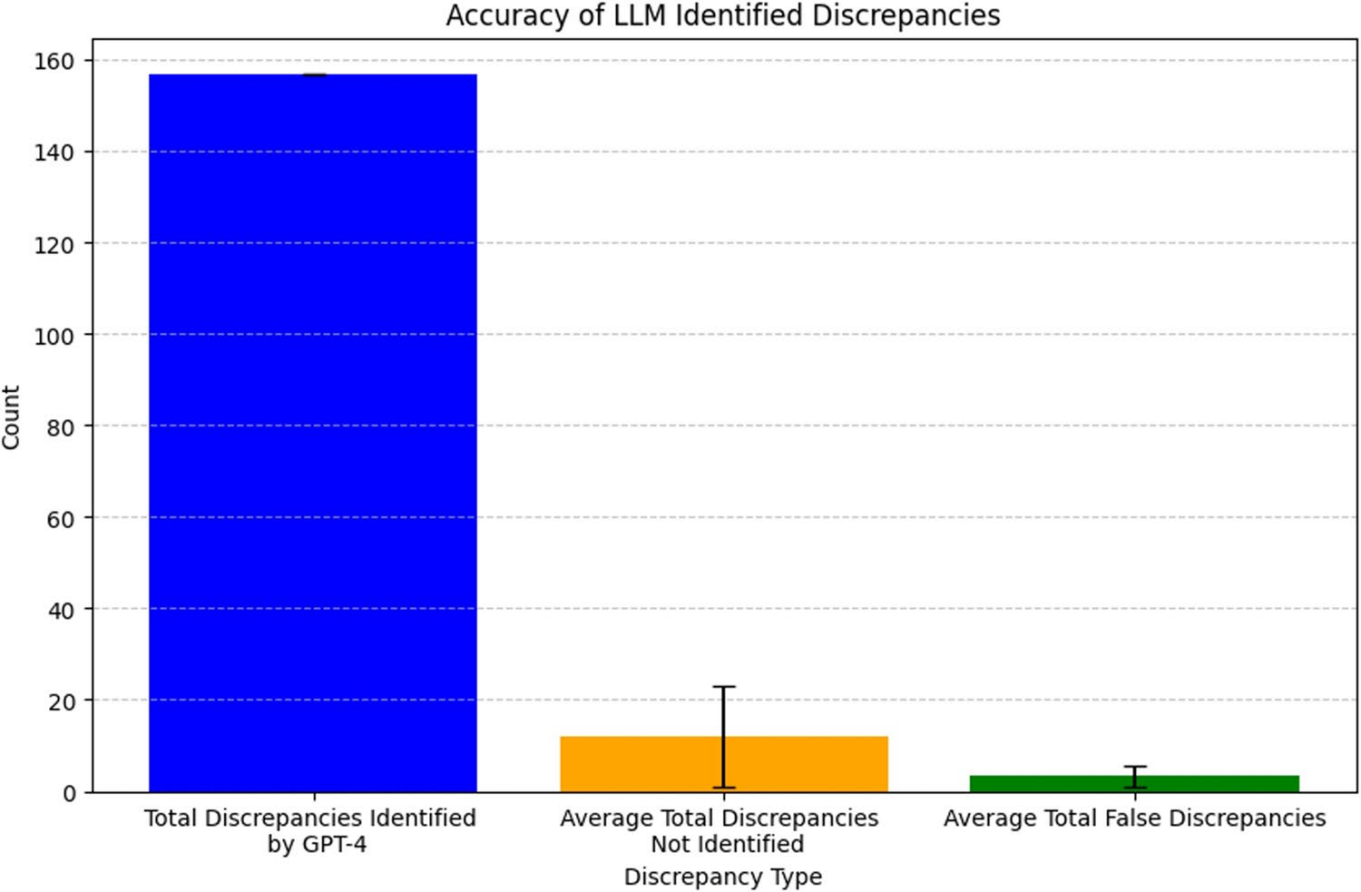
Bar plot demonstrating the total number of discrepancies identified by the LLM (left), average and standard deviation of the total number of discrepancies identified by radiologists and not identified by the LLM (center), and average and standard deviation of discrepancies identified by LLM which were described as falsely identified by radiologists (right)

For the synthetic neuroradiology reports, the LLM identified 149 total discrepancies and identified a higher average number of discrepancies (*μ* = 2.98, *σ* = 1.74) compared to human raters (*μ* = 2.43, *σ* = 1.70). A Wilcoxon signed-rank test showed that this increase was statistically significant (*W* = 19.0, *p* < 0.001). There was a moderately strong statistically significant positive correlation between discrepancies identified by AI and human raters (*r* = 0.778, *p* < 0.001, Fig. 5). Compared to the total number of identified discrepancies (Fig. 6), the average total unidentified discrepancies (i.e., false negative, *μ* = 12) and falsely identified discrepancies (i.e., false positive, *μ* = 3.3) were markedly lower. Most unidentified discrepancies (> 95%) and falsely identified discrepancies (80%) were identified by a single rater (Table 2). Upon retrospective interview, it was found that the rater had misinterpreted the instructions for the earlier cases. The data was retained in our analysis to avoid introducing selection bias.

**Table 2:** Number of discrepancies not identified by LLM (false negatives) and number of discrepancies falsely identified by LLM (false positives)

Rater	False negatives	False positives
A	2	1
B	0	1
C	34	8

### Discrepancy Severity and Type Classification

The LLM classified the majority of severities as minor (*n* = 67, 45%), followed by major (*n* = 38, 26%), stylistic (*n* = 22, 14%), with fewer classified as additions and other (Table 3). With respect to type classification, most discrepancies were classified as interpretive (*n* = 77, 52%) and perceptual (*n* = 42, 28%) with fewer typographic and other (Table 3).

**Table 3:** Distribution of discrepancies by severity and type as classified by the LLM

Category	Count, *n* (%)
Discrepancy severity	
Major	38 (26%)
Minor	67 (45%)
Stylistic	22 (14%)
Addition	20 (13%)
Other	2 (1%)
Discrepancy type	
Perceptual	42 (28%)
Interpretive	77 (52%)
Typographic	9 (6%)
Other	21 (14%)

Fleiss’ kappa for severity and type classifications were 0.346 and 0.340, respectively, and quadratic weighted kappa for severity was 0.622, indicating fair agreement among radiologists for exact severity and type and good overall agreement when accounting for the ordinal nature of severity. When combined with assessments by the AI, Fleiss’ kappa increased to 0.432 for severity (reflecting moderate agreement), 0.395 for type, and weighted kappa for severity increased to 0.669.

**Fig. 7:**
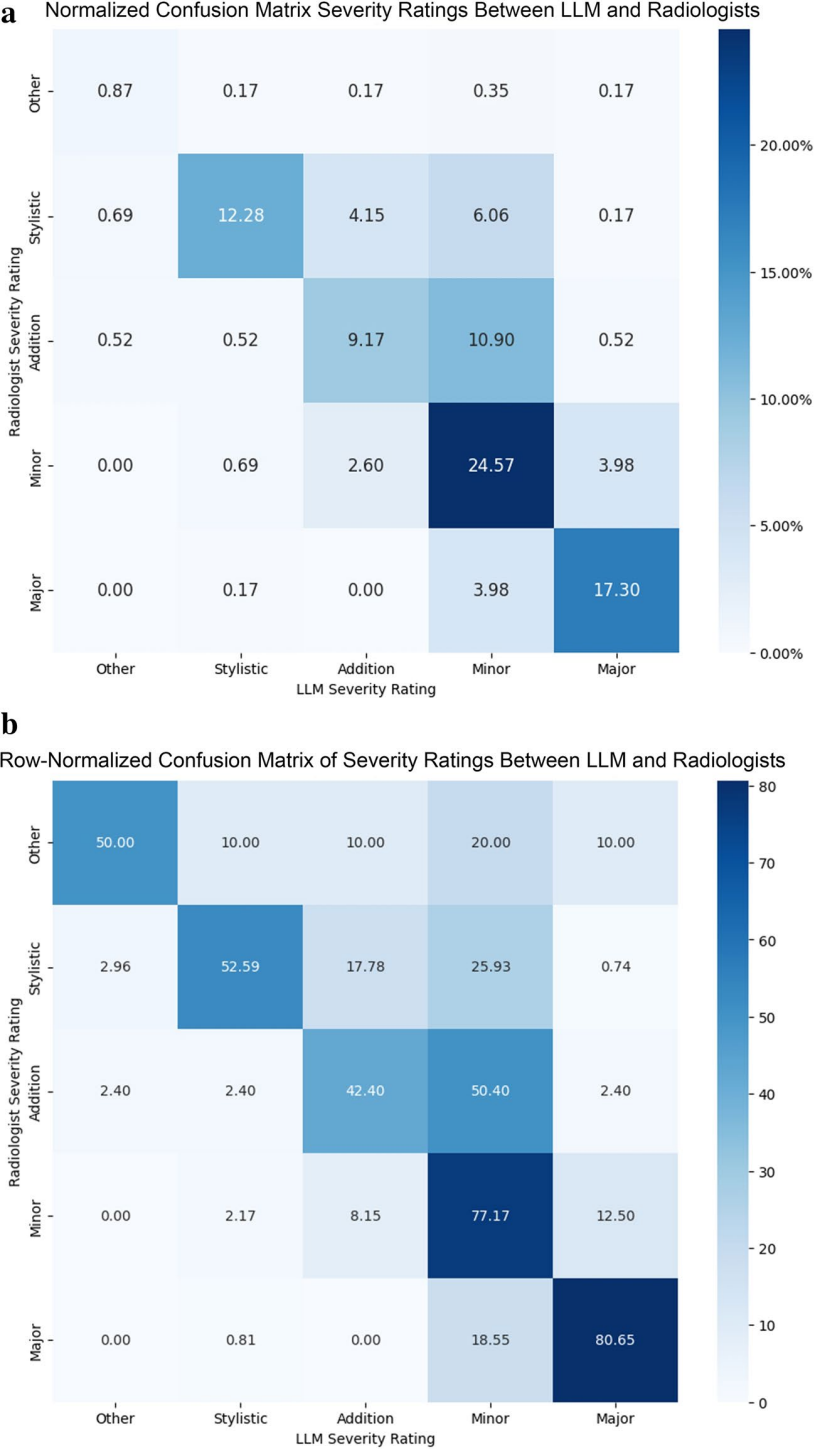
**a** Normalized confusion matrix demonstrating the percent frequency of discrepancy severity classifications by radiologists and LLM normalized over all samples. **b** Confusion matrix demonstrating the percent frequency of discrepancy severity classifications by radiologists and LLM normalized by row

**Fig. 8:**
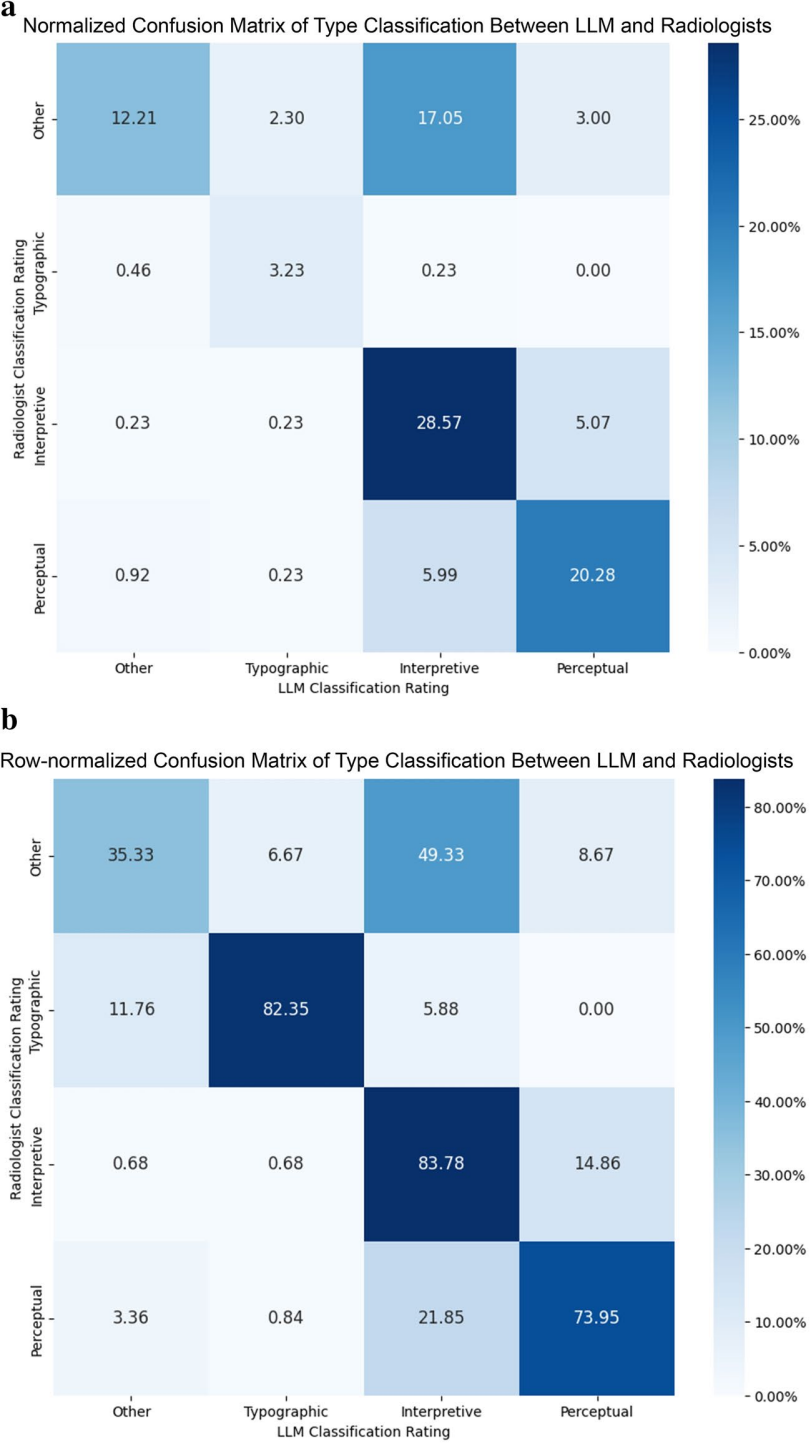
**a** Confusion matrix demonstrating the percent frequency of discrepancy category classification by radiologists and LLM normalized over all samples. **b** Row-normalized confusion matrix demonstrating the percent frequency of discrepancy category classification by radiologists and LLM

Radiologists agreed with the model’s assessment of discrepancy severity approximately 64% of the time (Fig. 7a). When ratings disagreed, the model was more likely to overestimate the severity of discrepancies. Severity classifications were most accurate when discrepancies were either major or minor (Fig. 7b; Table 3). Major discrepancies were the single group with the highest F1 score (0.78). Weighted overall F1 score was 0.66 (Table 4). The model and radiologists agreed with respect to type approximately 64% of the time as well (Fig. 8a). Perceptual errors represented the classification with the highest F1 score (0.72), and the weighted overall F1 score was 0.64 (Table 5).

**Table 4:** Precision, recall, and F1 score for LLM severity ratings compared to radiologist ratings

Class	Precision	Recall	F1 score
Other	0.38	0.50	0.43
Stylistic	0.89	0.53	0.66
Addition	0.56	0.42	0.48
Minor	0.53	0.77	0.63
Major	0.76	0.81	0.78
Weighted overall	0.69	0.66	0.66

Discrepancies which the model assessed as major in severity demonstrated the highest F1 score (0.78). The weighted overall F1 score was 0.66

**Table 5:** Precision, recall, and F1 score for model type classification compared to radiologists’ ratings

Class	Precision	Recall	F1 Score
Other	0.84	0.35	0.50
Typographic	0.52	0.82	0.64
Interpretive	0.54	0.84	0.66
Perceptual	0.71	0.74	0.72
Weighted overall	0.71	0.65	0.64

Discrepancies, which the model assessed as major in severity, demonstrated the highest F1 score (0.78). The weighted overall F1 score was 0.66

A focused analysis of model agreement was performed for discrepancies where there was uniform agreement between raters for severity and type (*n* = 57 and 59, respectively). The weighted overall F1 score was 0.95 for severity and 0.86 for type in these cases (Appendix C).

### RexVal Data

**Fig. 9:**
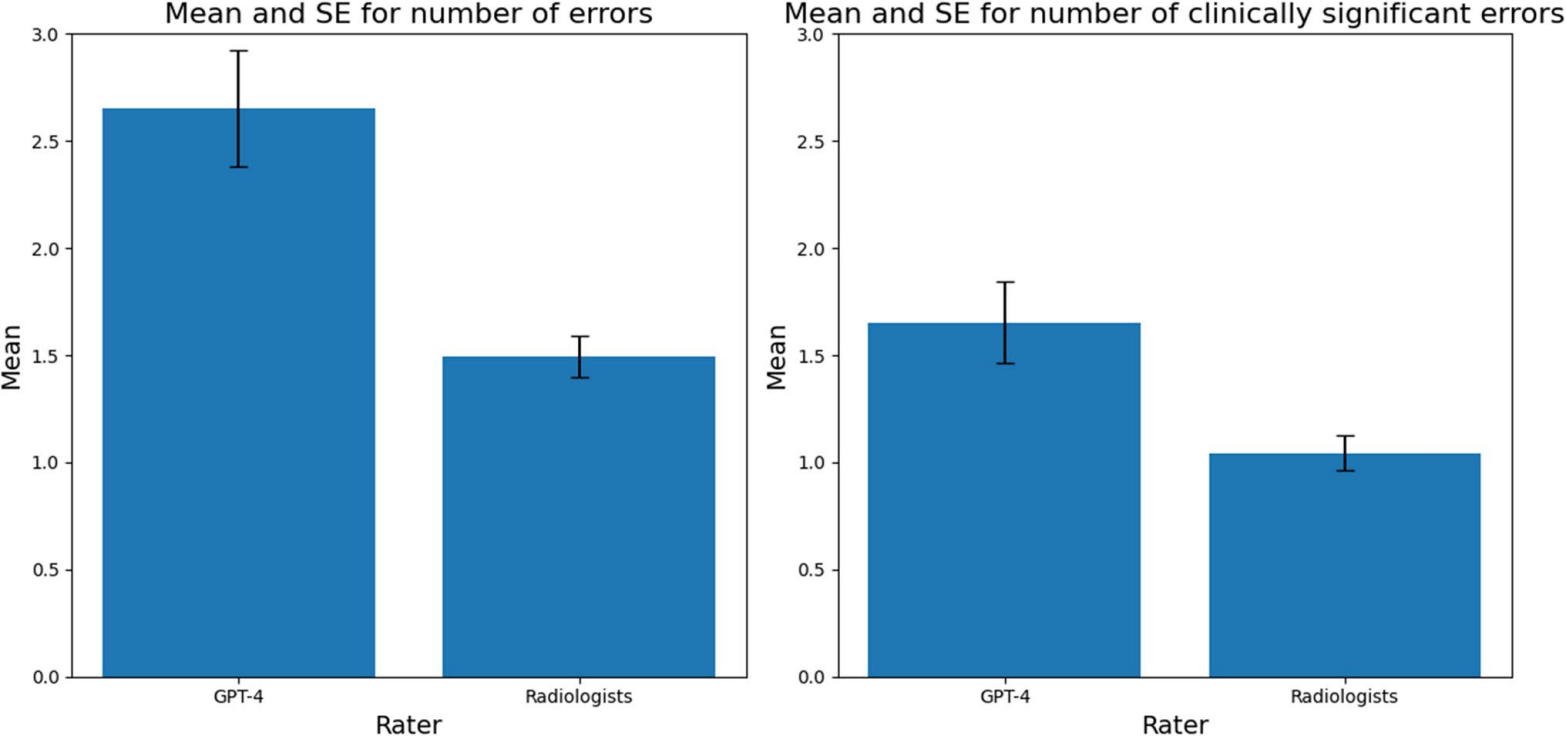
Mean number of overall discrepancies (left) and clinically significant discrepancies (right) identified by LLM compared to radiologists from the RexVal dataset. GPT-4 identified significantly more overall and clinically significant discrepancies compared to radiologists

**Fig. 10:**
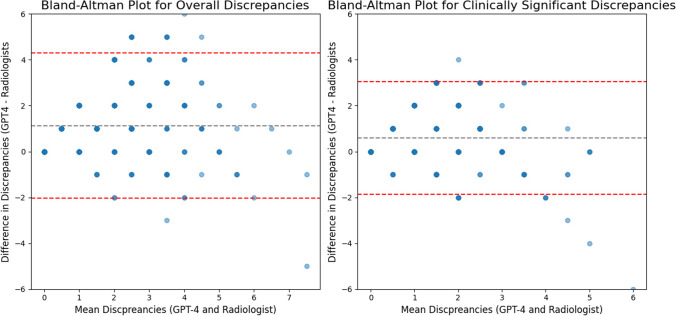
Bland–Altman plots demonstrating difference in discrepancies by mean discrepancies in overall discrepancies (left) and clinically significant discrepancies (right) identified by LLM and radiologists from the RexVal dataset

When evaluated on the RexVal dataset, our model's results were comparable to its performance on our synthesized dataset, identifying significantly more discrepancies than the expert annotators (Fig. 9) and showing a moderately strong positive correlation with their assessments (Fig. 10*r* = 0.588 for all discrepancies, *r* = 0.581 for clinically significant discrepancies, *p* < 0.001).

### Topics for Review

**Fig. 11:**
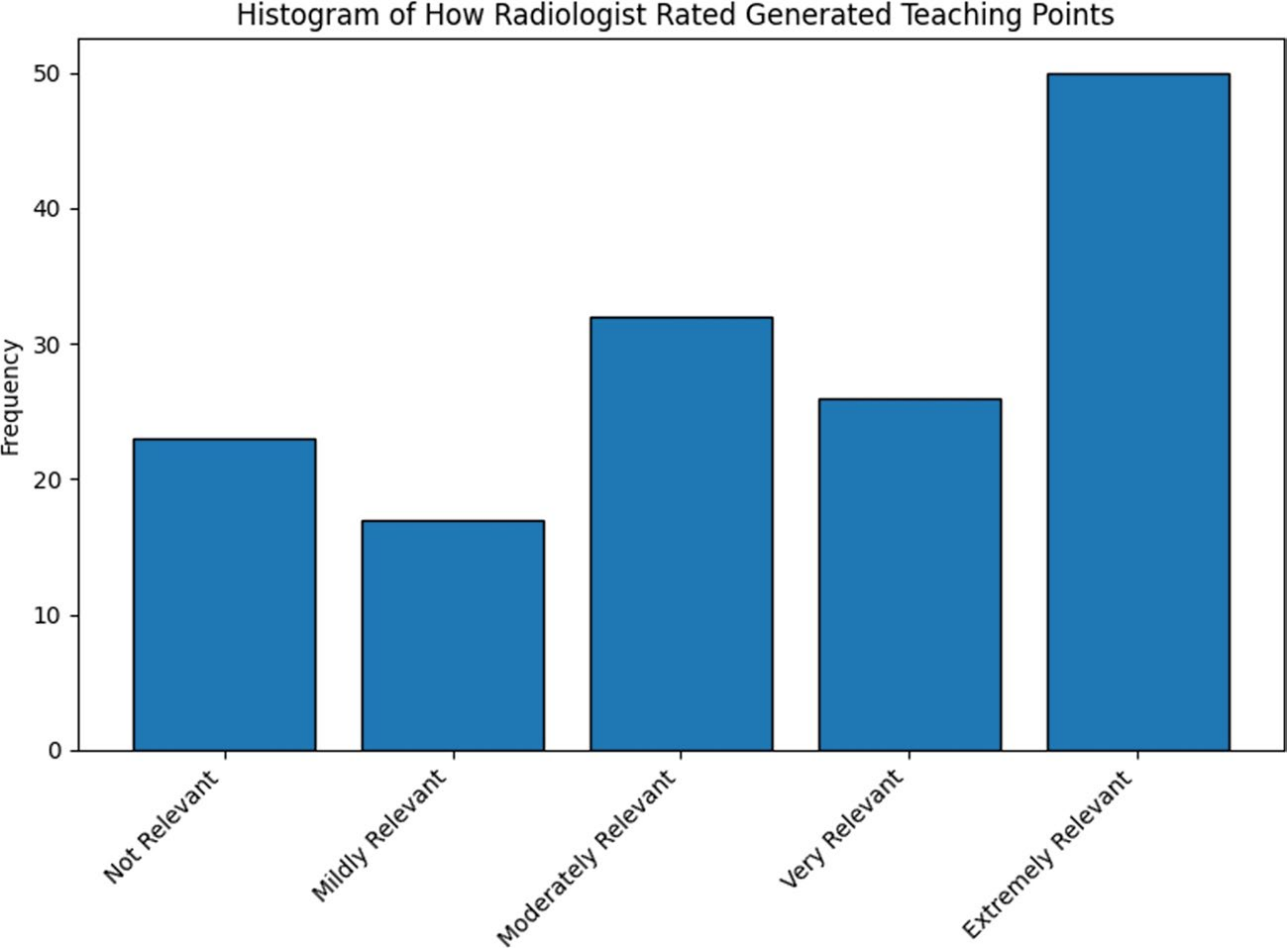
Histogram showing the distribution of radiologist ratings for the relevance of LLM-generated teaching points. A total of 84.5% of teaching points were considered relevant, with the majority being considered extremely relevant

**Fig. 12:**
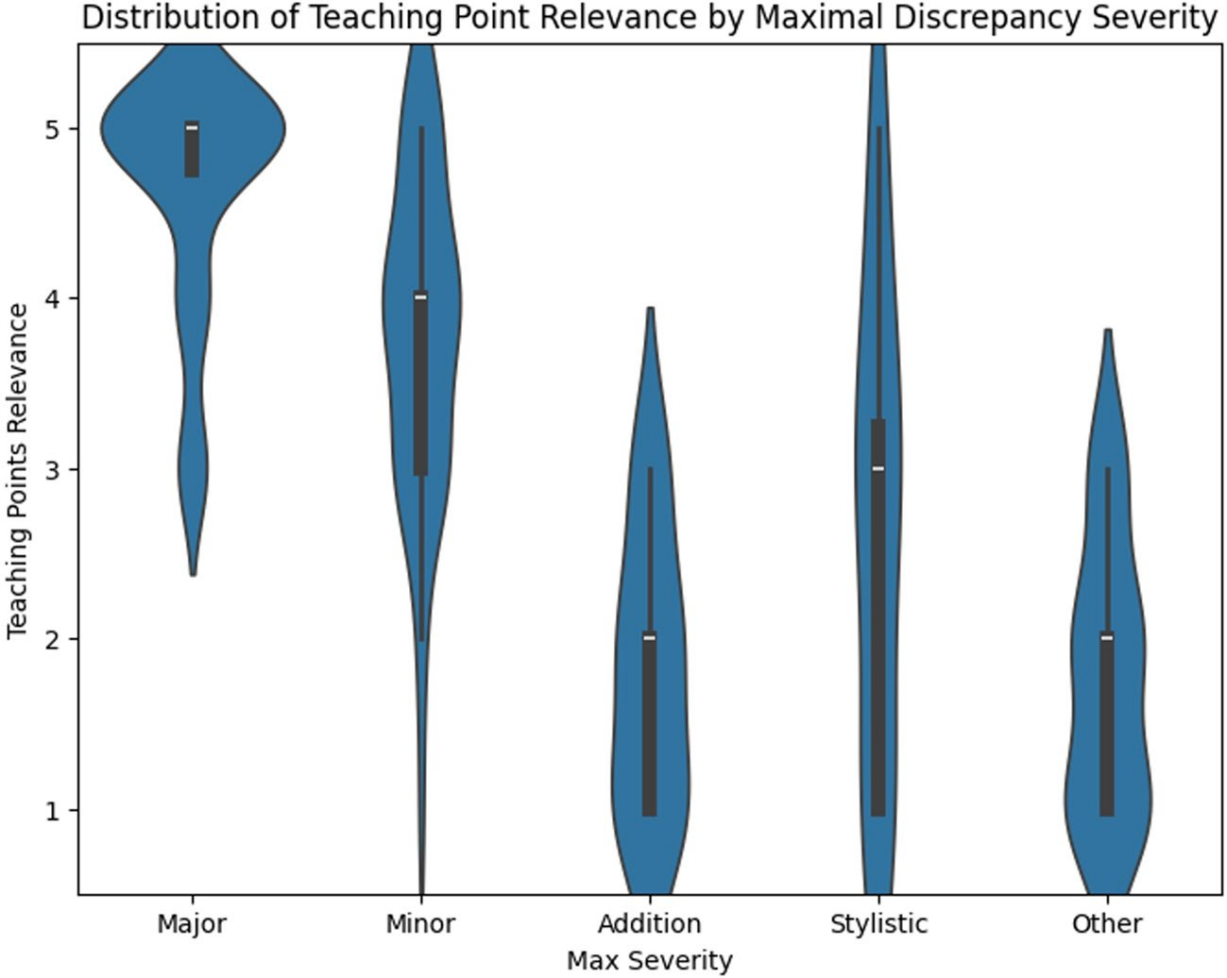
Violin plot demonstrating the distribution of assessed teaching point relevance by maximal discrepancy severity. Higher relevance of generated teaching points was associated with reports containing discrepancies of which the maximum severity was either major or minor

Expert radiologists indicated that the teaching points generated by the LLM were relevant in 84.5% of cases (Fig. 11). A Spearman’s correlation demonstrated that the degree of relevance was highly significantly related to the maximal severity of discrepancy identified in the case (*ρ* = 0.76, *p* < 0.001, Fig. 12).

### Reproducibility Analysis

Discrepancy detection and discrepancy severity classification were repeated 5 × , resulting in 600 samples for reproducibility analysis. The reproducibility was moderate to good (ICC (2,1) = 0.690, coefficient of variance = 0.35) for the number of discrepancies identified. For maximal discrepancy severity, reproducibility was substantial (Fleiss’ kappa = 0.718) and very good (weighted kappa = 0.94).

## Discussion

Our study demonstrates that LLMs can parse analogs of preliminary and finalized radiology reports, sensitively identify discrepancies, perform multilabel discrepancy classification with variability similar to radiologists and with good output reproducibility, and generate teaching points found relevant by expert radiologists.

### Sensitive Discrepancy Detection and Analysis

The LLM demonstrated exquisite sensitivity to discrepancies between paired analogs of preliminary and finalized radiology reports. This sensitivity of LLMs to small textual changes is well-known. In 2023, Anthropic boasted that Claude was able to identify a single line change in *The Great Gatsby* within 22 s [28]. The number of discrepancies identified was greater than and moderately positively correlated with discrepancies identified by expert radiologists. This phenomenon appears to be related to the method of parsing discrepancies. For example, a radiologist may consider alterations to the findings and impression to reflect a missed ischemic cerebral infarct with associated mass effect and communication of further steps as a single discrepancy. However, the LLM may consider this to represent multiple discrepancies, including (1) A missed ischemic cerebral infarct, (2) missed findings of mass effect, (3) changes to the overall impression, and (4) communication/recommendation of next steps. During our repeatability analysis, this also appeared to represent a source of variation in the number of discrepancies, as the quantification of discrepancies related to a single concept was inconsistent. Additionally, some of this variation may be related to how our study defined true discrepancies, considering stylistic discrepancies to be a type of true discrepancy. Depending on the intent, further studies may wish to utilize additional prompting techniques, chained or ensemble prompting techniques, or perhaps finetuning to constrain this multiplicity [29].

### Discrepancy Classification and Feedback

Expert radiologists mostly agreed with the LLM regarding classifications of discrepancy severity and type. We also found that the agreement of the LLM with radiologists was similar to the agreement between the radiologists themselves, as Fleiss’ and weighted kappa mildly increased with the addition of LLM ratings for both tasks. Weighted overall F1 scores on both classification tasks were moderate but markedly improved in cases where radiologists uniformly agreed on the classification of the discrepancy, reflecting high model performance in classifying more straightforward discrepancies. The highest overall agreement occurred when the discrepancy was considered major in severity and perceptual in nature. The agreement was also strong for minor severity discrepancies and discrepancies of interpretive or typographical nature. Discrepancy severities and categories with lower agreement (such as stylistic discrepancies or those classified as “other”) may be less reliable or valuable for potential data analytic or tracking purposes.

In cases where severity assessments were discordant, the LLM tended to overestimate the severity of discrepancies compared to expert radiologists. The mechanism of this overestimation is not entirely clear. One conjecture is that this may be related to the experts’ nuanced understanding of modifiers that may influence the clinical significance or potential harm associated with various discrepancies. For example, the LLM classified a discrepancy concerning a missed old rib fracture in the preliminary report as a major discrepancy, perhaps indicating that the model did not appropriately account for the age of the fracture. Tejani et al. [30] employed bidirectional language models with a finetuned classification layer and used a sequence classification explainer that identified the specific words that contributed the most to their model’s classification task. Employing similar techniques to explain model attention in future studies may be helpful. Additional strategies that could potentially result in improved classification could include additional prompting techniques, using an increased number of exemplars for few-shot prompting, prompting styles with increased reasoning capacity (e.g., tree-of-thought, recursion of thought, etc.), retrieval augmented generation (RAG), and finetuning. [29].

The LLM also demonstrated moderate to good consistency of outputs concerning the number of discrepancies identified and the exact discrepancy classification. When considering the ordinal nature of severity, the model’s reliability was very good, with a weighted kappa of 0.94, indicating that variation was more likely to occur between neighboring severities (e.g., major and minor) compared to distant severities (e.g., major and addition, stylistic, other, or none). This reliability was promoted by using a temperature setting of 0; however, alternative prompting techniques, ensembling, and finetuning could provide more reliable output.

The teaching points generated from the identified discrepancies were found to be relevant in 84.5% of cases, and the degree of relevance increased as the severity of discrepancies increased. This suggests that generating teaching points is most beneficial when severe discrepancies are identified, while the recommendations from low-severity discrepancies may be less useful. Restricting teaching point generation to reports containing minor or major discrepancies could be more valuable for trainees and reduce computational costs. We utilized a single prompt for teaching point generation; however, dynamically generated prompts based on study type and trainee history could also potentially generate more relevant teaching points.

### Limitations and Next Steps

Due to institutional limitations on data usage and lack of access to an institutionally approved GPT instance, we could not process real reports and were limited to synthesized and open-source datasets. These datasets were limited in subspecialties, and the distribution of discrepancy types was not explicitly balanced. Additionally, the RexVal dataset, while useful for evaluating discrepancy detection, contained only impressions and used auto-generated reports rather than actual trainee preliminary reports. Thus, the datasets employed may not fully represent the breadth and contents of real trainee-generated preliminary reports. Future research should prioritize evaluating real reports using protocols appropriate to their local institutions (perhaps using local large language models or approved instances of closed large language models).

We chose to evaluate model accuracy compared to current clinical assessment at our institution. Thus, we instructed raters to utilize their implicit understanding of severity terms at our institution and employed a probabilistic model of assessing agreement. This approach aimed to evaluate the realism of the model's performance compared to current clinical assessment, which is known to be subjective and variable. However, future work may wish to assess an alternative hypothesis concerning whether large language models could offer a *more* objective assessment than humans. In this case, future studies may employ stricter definitions, consensus rating adjudication, and repeated analyses for radiologists and the LLM.

We utilized single instances of zero-shot, one-shot, and few-shot prompts with additional techniques preliminarily tuned for task performance. While the model’s performance was promising, this approach may not represent the optimal prompting approach. Alternative prompting strategies (such as elaborate CoT prompting, decomposition, and ensembled prompts [29]), RAG, and fine-tuning may result in higher performance, and future studies would benefit from employing these techniques and assessing their comparative performance.

Radiologists and the LLM generally agreed on the report classification, and the LLM demonstrated good reliability. However, we would hesitate to suggest that this classification is reliable enough for formal trainee assessment. A separate limitation of this approach is the program’s sole reliance on textual data. The severity and other characteristics of a discrepancy may be intrinsically linked to the visual context of the images, which textual analysis alone cannot capture. For example, some attendings at our institution argue that the severity of a discrepancy is related to its obviousness relative to the trainee’s level of training. Some might argue that missing an obvious intracranial hemorrhage is more severe than a subtle intracranial hemorrhage in a motion-degraded study and that an error by a neuroradiology fellow could be considered more severe than an error by a less experienced trainee. Future implementation of multimodal models, incorporating both textual and visual data, and fine-tuning performance using real radiologic images and reports could enhance accuracy and reliability and potentially allow for increased educational capabilities (such as generating an image of the discrepancy or identifying relevant sequences or slices). However, this approach may be technically demanding, and its current feasibility is unclear [31].

An additional limitation of this study was the lack of resident feedback on the model’s output, including the generated teaching points. As this study focused primarily on assessing the accuracy of the model’s outputs, the output format was not optimized for trainee consumption. The lengthy output generated in our study may deter trainee usage, and a short summary may be more desirable. Our future work will prioritize optimizing the output for trainee viewing and evaluating its impact on their learning.

### Additional Future Applications

LLMs offer a promising solution for enhancing radiology education by automating the analysis of report discrepancies and providing trainees with efficient and comprehensive feedback. We are implementing and refining this system at our institution, incorporating findings from this study to optimize future versions. With these changes and the rapid advancements in LLM technology, we anticipate even greater performance and utility in subsequent iterations of this program.

Using LLMs, we can develop novel classifications of report discrepancies that go beyond severity or mechanisms of error. This could include classifications based on anatomic location, density/signal characteristics, patient factors, specific disease processes, or even the case’s complexity. This can potentially create an entirely new domain for understanding and addressing the mechanisms of radiologic error.

There is also substantial potential in accumulating this revision data over time. The data could be collated across multiple studies of the same trainee, multiple trainees of any cohort, multiple cohorts within a residency class, and even expanded to national and international scales. Comprehensive learning profiles for each group could be generated, allowing educators to offer more tailored feedback and perhaps revolutionizing personalized AI-augmented radiology education [18].

Finally, the factors that contribute to reduced “in-person” readouts are not expected to decrease in the near future. Methods of supplementing training and education will remain of extreme interest to radiology education.

## Conclusions

Our study demonstrates the significant potential of generative AI to enhance radiology education by automating the identification and classification of report discrepancies and generating relevant feedback. While further research is needed to optimize the interface between AI models and report revision data, the ability of generative AI to detect differences between preliminary and finalized reports and suggest relevant review topics boasts the potential for transformative impact and contribution to a new era of AI-augmented precision learning.

## Supplementary Information

Below is the link to the electronic supplementary material.

Supplementary file1 (PDF 96 KB)

Supplementary file2 (CSV 696 KB)

Supplementary file3 (PDF 139 KB)

## Data Availability

The data from this study, including the prompts used and the model outputs generated, are available as supplemental material accompanying this publication. Rating data and code used in this study can be provided by the corresponding author upon reasonable request.
