# Supplementary material for: From Revisions to Insights: Converting Radiology Report Revisions into Actionable Educational Feedback Using Generative AI Models
Source: J Imaging Inform Med. 2024 Aug 19;38(2):1265–79. doi: 10.1007/s10278-024-01233-4 (PMC11950553; doi:10.1007/s10278-024-01233-4)
Supplement: Supplementary file 1 — Supplementary file1 (PDF 96 KB) [file 10278_2024_1233_MOESM1_ESM.pdf]

Appendix A. Prompts:

1. Discrepancy Detection

Prompt

```
$discrepanciesPrompt = "You are an academic radiologist and have produced a finalized report ($final) from a preliminary report created by a trainee ($preliminary). If present, please describe the significant unique discrepancies between the finalized report and the preliminary report. Please make sure that the discrepancies are actual discrepancies between the content of the report and not merely discrepancies in position within the report. These discrepancies should be unique and there should not be multiple of the same discrepancy. Related discrepancies should be combined into a single discrepancy. Please post a final line with the number of discrepancies in the following format: Discrepancies: #. This separate line should be by itself without a header.";
```

Techniques employed:

Prompt Segment	Technique
1. You are an academic radiologist	Role Instruction
2. and have produced a finalized report ($final) from a preliminary report created by a trainee ($preliminary).	Context instruction
3. If present, please describe the significant unique discrepancies between the finalized report and the preliminary report.	Zero-shot task.
4. Please make sure that the discrepancies are actual discrepancies between the content of the report and not merely discrepancies in position within the report. These discrepancies should be unique and there should not be multiple of the same discrepancy. Related discrepancies should be combined into a single discrepancy.	Conditional instruction
5. Please post a final line with the number of discrepancies in the following format: Discrepancies: #. This separate line should be by itself without a header.";	Output instruction.

2. Discrepancy Severity Classification

Prompt

```
$scoringDiscrepanciesDegreePrompt = "You are an academic radiologist and you are given the following analysis of discrepancies ($discrepanciesOutput) between a trainee's preliminary report and a finalized report. For each discrepancy, please identify and explain whether the missed findings in trainee's preliminary report is major (could cause immediate or irreparable harm to patients such as missed pneumothorax, pulmonary embolism, acute intracranial hemorrhage, large vessel occlusion, bowel perforation, etc.), minor (changes that may affect clinical treatment but without significant potential for immediate harm such as favoring a different etiology within a list of differentials, etc.), additions (changes that add more detail but do not affect clinical treatment), stylistic changes (which do not change the content but only the phrasing) or other. Please also post a comma-delimited line listing the major discrepancies in the following format: Major discrepancies: a short description of discrepancy (or none if none are present). Please also post a final line with number of each type of error in the following format: Major: #, Minor:#, Additions:#, Stylistic:#, Other:#. This separate line should be by itself without a header. If there are no discrepancies, do not create hypotheticals."
```

Techniques employed:

Prompt Segment	Technique
1. You are an academic radiologist	Role Instruction
2. and you are given the following analysis of discrepancies ($discrepanciesOutput) between a trainee's preliminary report and a finalized report.	Context instruction
3. For each discrepancy, please identify and explain	Thought generation/Zero-shot Chain of thought*
4. whether the missed findings in trainee's preliminary report is<br><br>major (could cause immediate or irreparable harm to patients such as missed pneumothorax, pulmonary embolism, acute intracranial hemorrhage, large vessel occlusion, bowel perforation, etc.),<br><br>minor (changes that may affect clinical treatment but without significant potential for immediate harm such as favoring a different etiology within a list of differentials, etc.),<br><br>additions (changes that add more detail but do not affect clinical treatment), stylistic changes (which do not change the content but only the phrasing) or other.	Hybrid task including<br><br>Few-shot<br><br>One-shot<br><br>Zero-shot
5. Please also post a comma-delimited line listing the major discrepancies in the following format: Major discrepancies: a short description of discrepancy (or none if none are present). Please also post a final line with number of each type of error in the following format: Major: #, Minor:#, Additions:#, Stylistic:#, Other:#. This separate line should be by itself without a header.	Output instruction
6. If there are no discrepancies, do not create hypotheticals.	Conditional instruction.

3. Discrepancy Type Classification

Prompt

```
$scoringDiscrepanciesTypePrompt="You are an academic radiologist and you are given the following
analysis of discrepancies ($discrepanciesOutput) between a trainee's preliminary report and a
finalized report. For each discrepancy, please identify whether any discrepancies are likely
attributed to perceptual error (not seeing the finding), interpretive error (incorrectly
understanding a finding that was seen), typographical or other. Please post a final line with number
of each type of error in the following format: Perceptual: #, Interpretive: #, Typographic:#,
Other:#. This separate line should be by itself without a header. If there are no discrepancies, do
not create hypotheticals."
```

Techniques employed:

Prompt Segment	Technique
1. You are an academic radiologist	Role Instruction
2. and you are given the following analysis of discrepancies ($discrepanciesOutput) between a trainee's preliminary report and a finalized report.	Context instruction
3. For each discrepancy, please identify whether any discrepancies are likely attributed to perceptual error (not seeing the finding), interpretive error (incorrectly understanding a finding that was seen), typographical or other.	Zero-shot task.
4. Please post a final line with number of each type of error in the following format: Perceptual: #, Interpretive: #, Typographic:#, Other:#. This separate line should be by itself without a header.	Output instruction
5. If there are no discrepancies, do not create hypotheticals."	Conditional instruction.

4. Teaching Points Generation

Prompt

```
$scoringDiscrepanciesTeachingPointsPrompt = "You are an academic radiologist and you are given the following analysis of discrepancies ($discrepanciesOutput) between a trainee's preliminary report and a finalized report. The finalized report is ($final). Identify  topics in anatomy or pathology would be beneficial for the trainee to review based on the discrepancies. Please describe teaching points in detail giving relevant explanations for trainees. Please post a final comma delimited line with tagged anatomic or pathologic topics for review in the following format: Topics for review: topic 1, topic 2, etc. If there are no relevant topics to review based discrepancies, then say Topics for review:none. If there are no discrepancies, do not create hypotheticals.";
```

Techniques employed:

Prompt Segment	Technique
1. You are an academic radiologist	Role Instruction
2. and you are given the following analysis of discrepancies ($discrepanciesOutput) between a trainee's preliminary report and a finalized report. The finalized report is ($final).	Context instruction
3. Identify topics in anatomy or pathology would be beneficial for the trainee to review based on the discrepancies.	Zero-shot task.
4. Please describe teaching points in detail giving relevant explanations for trainees.	Generation/Zero-shot Chain of thought*
5. Please post a final comma delimited line with tagged anatomic or pathologic topics for review in the following format: Topics for review: topic 1, topic 2, etc.	Output instruction.
6. If there are no discrepancies, do not create hypotheticals.	Conditional instruction

5. Synthesized Neuroradiology Reports

Prompt

```
$reportGenerationPrompt = "I am making a program to analyze revisions in radiology reports. Please
generate a sample preliminary neuroradiology report and a sample finalized version of that report.
The type of examination and body part of the exam should be random. The report should randomly have
no significant change or discrepancies which are major (could cause immediate or irreparable harm to
patients such as missed pneumothorax, pulmonary embolism, acute intracranial hemorrhage, large
vessel occlusion, bowel perforation, etc.), minor (changes that may affect clinical treatment but
without significant potential for immediate harm such as favoring a different etiology within a list
of differentials, etc.), additions (changes that add more detail but do not affect clinical
treatment), stylistic changes (which do not change the content but only the phrasing) or other."
```

Techniques employed:

Prompt Segment	Technique
1. I am making a program to analyze revisions in radiology reports.	Context Instruction
2. Please generate a sample preliminary neuroradiology report and a sample finalized version of that report.	Zero-shot task.
3. The report should randomly have no significant change or discrepancies which are major (could cause immediate or irreparable harm to patients such as missed pneumothorax, pulmonary embolism, acute intracranial hemorrhage, large vessel occlusion, bowel perforation, etc.), minor (changes that may affect clinical treatment but without significant potential for immediate harm such as favoring a different etiology within a list of differentials, etc.), additions (changes that add more detail but do not affect clinical treatment), stylistic changes (which do not change the content but only the phrasing) or other.	Additional information/context
