# Supplementary material for: From Revisions to Insights: Converting Radiology Report Revisions into Actionable Educational Feedback Using Generative AI Models
Source: J Imaging Inform Med. 2024 Aug 19;38(2):1265–79. doi: 10.1007/s10278-024-01233-4 (PMC11950553; doi:10.1007/s10278-024-01233-4)
Supplement: Supplementary file 3 — Supplementary file3 (PDF 139 KB) [file 10278_2024_1233_MOESM3_ESM.pdf]

Appendix C. Model performance in cases with Uniform Radiologist Agreement

Severity

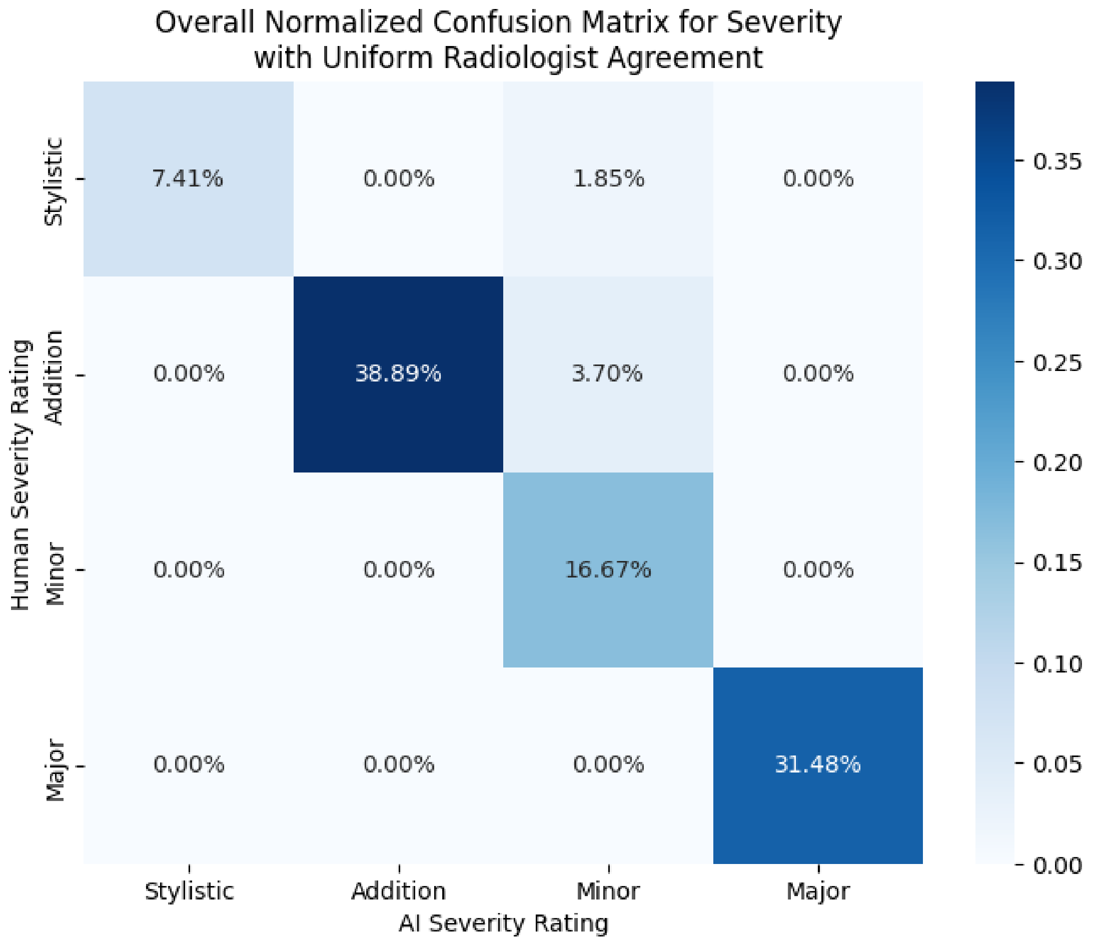

Class	Precision	Recall	F1 Score
Stylistic	1.00	1.00	1.00
Addition	1.00	0.80	0.89
Minor	0.75	1.00	0.86
Major	1.00	0.91	0.95
Weighted Overall	0.96	0.94	0.95

In discrepancies

Type

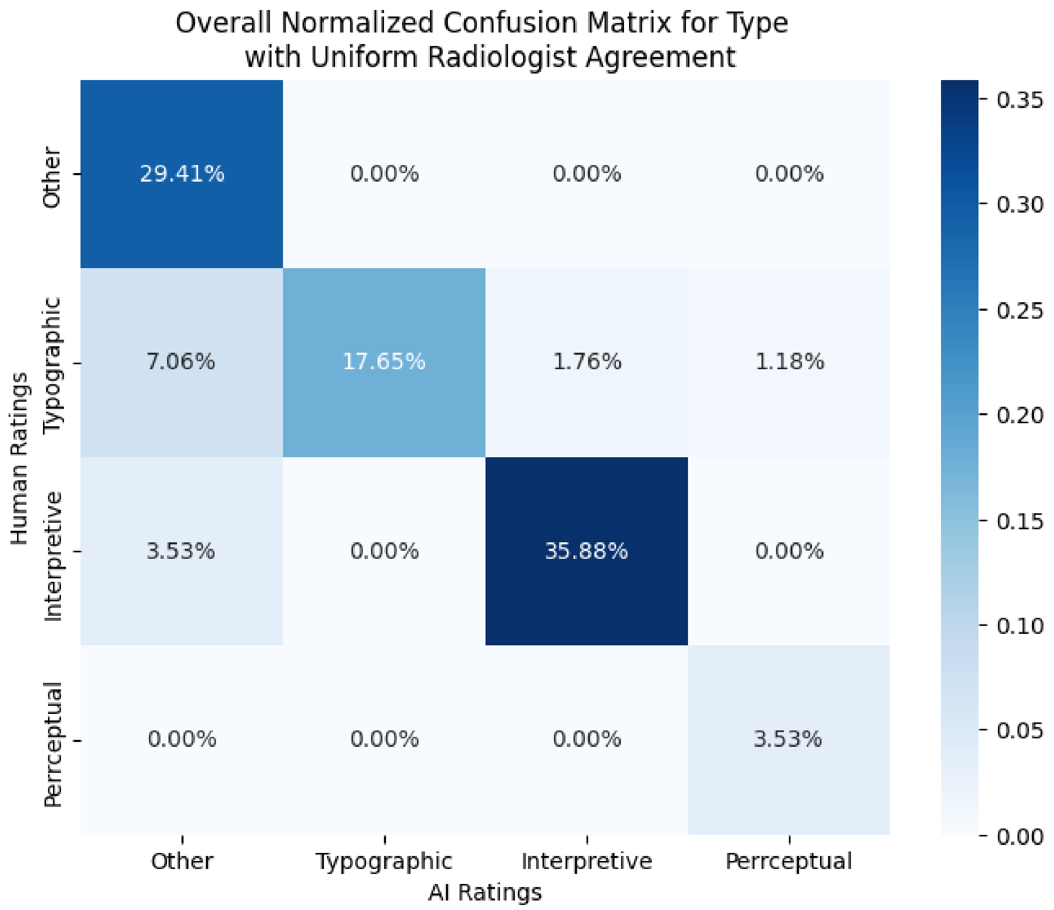

**Class**	**Precision**	**Recall**	**F1 Score**
Other	1.00	0.64	0.78
Typographic	0.75	1.00	0.86
Interpretive	0.74	1.00	0.85
Perceptual	0.95	0.91	0.93
Weighted Overall	0.89	0.86	0.86
